# Off-target pharmacological profiling of synthetic cannabinoid receptor agonists including AMB-FUBINACA, CUMYL-PINACA, PB-22, and XLR-11

**DOI:** 10.3389/fpsyt.2022.1048836

**Published:** 2022-12-15

**Authors:** Richard C. Kevin, Elizabeth A. Cairns, Rochelle Boyd, Jonathon C. Arnold, Michael T. Bowen, Iain S. McGregor, Samuel D. Banister

**Affiliations:** ^1^The Lambert Initiative for Cannabinoid Therapeutics, The University of Sydney, Camperdown, NSW, Australia; ^2^School of Pharmacy, The University of Sydney, Camperdown, NSW, Australia; ^3^School of Psychology, The University of Sydney, Camperdown, NSW, Australia; ^4^Brain and Mind Centre, The University of Sydney, Camperdown, NSW, Australia; ^5^School of Chemistry, The University of Sydney, Camperdown, NSW, Australia

**Keywords:** cannabinoid, synthetic, SCRA, G protein coupled receptor (GPCR), AMB-FUBINACA, off-target, toxicity

## Abstract

**Introduction:**

Synthetic cannabinoid receptor agonists (SCRAs) are a diverse class of new psychoactive substances that have been associated with multiple instances and types of toxicity. Some SCRAs appear to carry a greater toxicological burden than others, or compared to the prototypical cannabis-derived agonist Δ^9^-tetrahydrocannabinol (Δ^9^-THC), despite a common primary mechanism of action *via* cannabinoid 1 (CB1) receptors. “Off-target” (i.e., non-CB1 receptor) effects could underpin this differential toxicity, although there are limited data around the activity of SCRAs at such targets.

**Methods:**

A selection of 7 SCRAs (AMB-FUBINACA, XLR11, PB-22, AKB-48, AB-CHMINICA, CUMYL-PINACA, and 4F-MDMB-BUTINACA), representing several distinct chemotypes and toxicological profiles, underwent a 30 μM single-point screen against 241 G protein-coupled receptor (GPCR) targets in antagonist and agonist mode using a cellular β-arrestin recruitment assay. Strong screening “hits” at specific GPCRs were followed up in detail using concentration-response assays with AMB-FUBINACA, a SCRA with a particularly notable history of toxicological liability.

**Results:**

The single-point screen yielded few hits in agonist mode for any compound aside from CB1 and CB2 receptors, but many hits in antagonist mode, including a range of chemokine receptors, the oxytocin receptor, and histamine receptors. Concentration-response experiments showed that AMB-FUBINACA inhibited most off-targets only at the highest 30 μM concentration, with inhibition of only a small subset of targets, including H_1_ histamine and α_2B_ adrenergic receptors, at lower concentrations (≥1 μM). AMB-FUBINACA also produced concentration-dependent CB1 receptor signaling disruption at concentrations higher than 1 μM, but did not produce overt cytotoxicity beyond CP55,940 or Δ^9^-THC in CB1 expressing cells.

**Discussion:**

These results suggest that while some “off-targets” could possibly contribute to the SCRA toxidrome, particularly at high concentrations, CB1-mediated cellular dysfunction provides support for hypotheses concerning on-target, rather than off-target, toxicity. Further investigation of non-GPCR off-targets is warranted.

## Introduction

Synthetic cannabinoid receptor agonists (SCRAs) are a diverse and growing class of compounds that bind and activate cannabinoid 1 (CB1) and 2 (CB2) receptors (1, 2). Activation of CB1 receptors in the brain produces psychoactive effects, including the typical “high” associated with cannabis intoxication *via* the well-known cannabinoid receptor agonist, Δ^9^-tetrahydrocannabinol (Δ^9^-THC) (3). The earliest SCRAs were designed as research tools (4–6), but have since been used and abused for their CB1 receptor-mediated intoxicating effects, and are now the largest class of new psychoactive substances (NPS) (7).

In many countries, legislative controls have been used to limit access to SCRAs (8). However, SCRA manufacturers regularly develop new structural analogs to evade legislation and forensic detection (9). This has led to the proliferation of large numbers of novel SCRAs in NPS markets, which at the time of first detection are usually uncharacterised in terms of their pharmacological or toxicological properties. Subsequent pharmacological assessment has revealed a general trend toward increasing potency and efficacy at CB1 receptors over the past decade (10–13).

Although Δ^9^-THC is generally well-tolerated in humans, SCRAs are associated with an array of serious adverse effects (14). SCRAs have been associated with multiple instances and types of toxicity, ranging from transient adverse effects like anxiety, paranoia, and catalepsy, to severe and potentially long-lasting adverse effects like cardiac and kidney injuries, encephalopathy, coma, and even death (15–17). Notable instances of SCRA toxicity include a mass hospitalization event in New York, linked to AMB-FUBINACA (18), as well as fatal intoxications linked to this compound in New Zealand (19), poisonings linked to XLR-11 and associated *in vitro* nephrotoxicity (20, 21), and a range of poisonings that occurred in Russia linked to AB-CHMINACA, MDMB-CHMICA, and AB-PINACA, among others (22).

Curiously, some SCRAs appear to carry a significantly higher toxicological burden than others, or compared to Δ^9^-THC, despite a common and presumed primary mechanism of action *via* CB1 receptors. Potential explanations include extremely high CB1 receptor efficacy of some SCRAs (compared to partial agonists like Δ^9^-THC), biased signaling (23), formation of potentially toxic metabolites, thermolytic degradation of compounds when smoked (24, 25), or off-target effects.

Appreciable SCRA off-target activity might be expected given the large degree of structural diversity present within this class. For example, several first-generation aminoalkylindole SCRAs, including JWH-018 and AM-2201, are weakly potent but highly efficacious 5-HT_2B_ antagonists, while PB-22 is a 5-HT_2A_ agonist. A number of SCRAs including MDMB-CHMINACA, UR-144, STS-135, and others are GPR55 antagonists (26), while AM-2201 interacts with 5-HT_6_, and MDMB-CHMICA, MDMB-CHMINACA, MO-CHMINACA, MDMB-CHMCZCA, and others are micromolar inhibitors of GPR18 (27).

These data clearly show the potential for off-target SCRA effects, but to date, little systematic, broad screening has been conducted. Several first-generation SCRAs have been assessed for binding affinity at dopamine, GABA_A_, histamine, acetylcholine muscarinic, norepinephrine, opioid, serotonin, and sigma receptors, with modest binding and antagonist functional activity observed at 5-HT_2B_ (28). However, second and third generation SCRAs have since emerged in the NPS market, some associated with severe toxidromes beyond that of earlier compounds. Moreover, there are few data concerning SCRA binding and activity at targets beyond these initially assessed systems. To address this knowledge gap, we systematically assessed a selection of SCRAs for off-target effects using a broad, single point screen against a range of G protein-coupled receptors (GPCRs), for both agonist and antagonist functional activity.

## Materials and methods

### PathHunter β-Arrestin assay

XLR11, PB-22, AKB-48, AB-CHMINACA, CUMYL-PINACA, 4F-MDMB-BUTINACA (Caymen Chemical, Ann Arbor, MI) and AMB-FUBINACA (Cerilliant, Round Rock, TX) were screened for β-arrestin activity using the PathHunter system in the gpcrMAX and orphanMAX assay panels (performed by Eurofins Discovery Services–DiscoverX, Freemont, CA, USA). Assays were performed using standard conditions, in technical replicate at 30 μM to minimize false negatives. For the gpcrMAX panel, control concentration-response curves with known agonists were generated simultaneously and used to normalize all other data to the maximal and vehicle-only response (1% DMSO). For orphanMAX, assay positive and negative controls were also run simultaneously with each receptor, using the enzyme fragment complementation controls against the β-gal ProLink peptide-tagged GPCR. Cells were pre-incubated with the compound for 30 min in antagonist mode (EC_80_ agonist), or 2–4 h in agonist (including orphan) mode. Cell types, incubation time, control agonists, and EC_80_ for each target has been included in Supplementary Table 1. All assays met quality control criteria, including RC50 and signal-to-background aligning to historical average, and Z' >0.5.

Activity, or a “hit,” was defined as >30% in agonist mode, >35% in antagonist mode, or >50% in the orphan screen (agonist mode). Where these criteria were met, signal-to-noise (S/N) and coefficient of variation (CoV) were also calculated, defined as:


$$\begin{array}{l} \begin{array}{ccc} \; & Agonist\;mode\; & Antagonist\;mode\\ S/N\; & \frac{Compound\;mean}{Baseline\;mean+\left(Baseline\;SD\times 3\right)}\; & \frac{EC_{80}\;mean}{Compound-\left(Compound\;SD\times \;3\right)}\\ CoV\; & \frac{Baseline\;mean}{Compound\;mean-\left(Compound\;SD\times \;3\right)}\; & \frac{Compound\;mean}{EC_{80}\;mean-\left(EC_{80}\;SD\;\times \;3\right)} \end{array} \end{array}$$

Hits were verified to have S/N>1 and a CoV <1. Thresholds and subsequent validation were guided by Eurofins. Descriptive data (means ± S.E.M) was generated in Graphpad Prism (version 9.2.0, San Diego, CA).

### Follow-up concentration-response assays

Follow up experiments were prioritized based on the difference between S/N and CoV. Targets with a >5-fold difference between S/N and CoV were investigated for their potential contribution to the AMB-FUBINACA toxidrome with 10-point concentration-response curves generated in technical replicate (range 1.5–30 μM) using the EC_80_ of the relevant agonist, where applicable in antagonist mode. Simultaneous agonist and antagonist curves were run in parallel to ensure reliability of the assay (see Supplementary Table 2), with both RC_50_ and signal to background aligning with historical averages, and Z' >0.5. RC_50_ was estimated using a four-parameter fit (CBIS Data Analysis Suite, ChemInnovation, San Diego, CA) on normalized data.

### AMB-FUBINACA cytotoxicity assay

AtT20 cells expressing either human CB1 (hCB_1_) or CB2 (hCB_2_) receptors or wildtype (WT) were grown to confluence in T75 cm^2^ flasks in Dulbecco's modified eagle medium containing 10% fetal bovine serum, 100 U penicillin/streptomycin, and 80 μg/mL hygromicyin (Sigma-Aldrich/Merck; Castle Hill, NSW, Australia). Cells were plated in 96-well-microplates at 30,000 cells per well and allowed to adhere overnight in 5% CO_2_. The following morning, cells were treated with either vehicle (0.1% DMSO; negative control), 30 μM Δ^9^-THC, or 30 μM AMB-FUBINACA. At 1 or 6 h, the amount of lactate dehydrogenase in the cell media was tested, and compared with positive control (lysed cells) as per manufacturer's instructions (Dojindo Molecular Technologies, Inc.; Rockville, MD, USA), with readings performed on a Clariostar Plus plate reader (BMG Labtech, Mornington, VIC, Australia). Experiments were performed in triplicate. In order to measure any accumulating LDH, media was not changed after initial incubation. Data is represented as means ± S.E.M. A two-way ANOVA with was performed on the data at each time point, with Tukey's multiple comparison test (α = 0.05). A *p* < 0.05 was considered statistically significant.

## Results

### PathHunter β-arrestin assay

β-arrestin recruitment is an important step in the regulation of GPCR and G protein signaling, and can be probed as a measure of GPCR activation across a wide range of receptors (29). The PathHunter β-arrestin assay was performed in both agonist and antagonist modes across 168 GPCRs, and in agonist mode in 73 orphan GPCRs.

**Figure 1:**
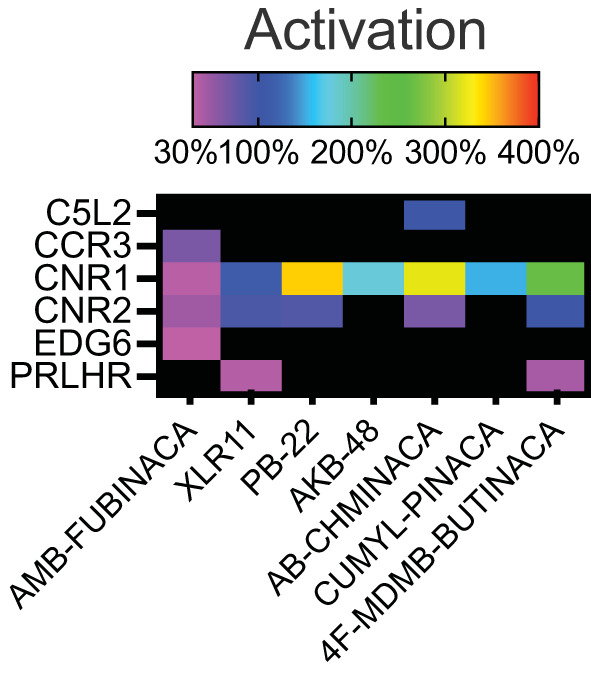
Activity of the SCRAs AMB-FUBINACA, XLR11, PB-22, AKB-48, AB-CHMINICA, CUMYL-PINACA, and 4F-MDMB-BUTINACA at 30 μM in a β-arrestin recruitment screen. Heatmap depicting targets where above-threshold activation (>30%) was reached with at least one SCRA. The full list of targets screened is included as Supplementary Table 1.

**Figure 2:**
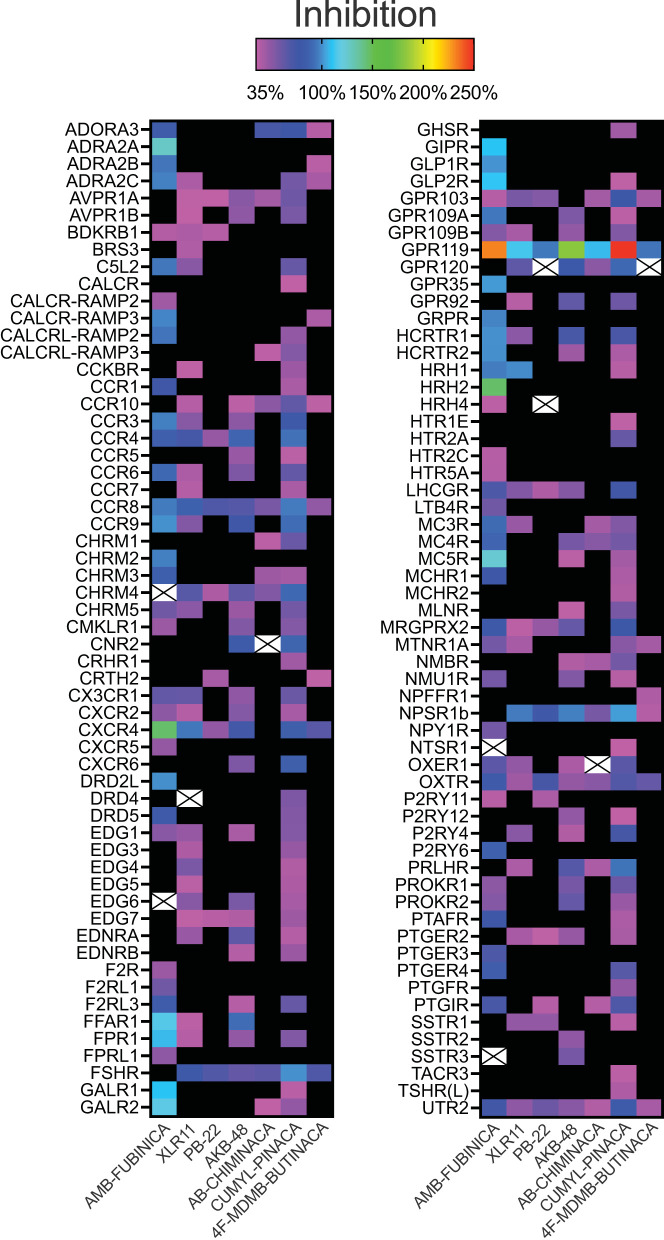
Inhibitory activity of the SCRAs AMB-FUBINACA, XLR11, PB-22, AKB-48, AB-CHMINICA, CUMYL-PINACA, and 4F-MDMB-BUTINACA at 30 μM in a β-arrestin recruitment screen. Heatmap depicting targets where above-threshold inhibition (>35%) was reached with at least one SCRA. Crosses indicate where antagonism was >35% but had S/N<1 or CoV>1. The full list of targets screened is included as Supplementary Table 1.

Activation above threshold (>30%) was elicited by SCRA compounds in very few targets, primarily at CB1 and CB2, and at no orphan GPCRs (threshold >50%; Figure 1). However, inhibition (>35%) occurred across a wide range of non-orphan GPCR targets (range 16–86 off-targets per compound; Figure 2).

AMB-FUBINACA and CUMYL-PINACA inhibited the greatest number of targets above 35% threshold (Figure 2), with AMB-FUBINACA generally inhibiting targets to a greater extent (mean inhibition = 81.16; 3.80%), whereas 4F-MDMB-BUTINACA and PB-22 inhibited the fewest off-targets (*n* = 16 and 20, respectively). Ten hits failed to meet S/N and/or CoV cut-offs (mean inhibition = 44.81 ± 1.85%), and were excluded from further analysis.

Sixteen targets were engaged by 5 or more of the screened compounds, including activation at CB1 and CB2, and inhibition at AVPR1A, CCR4, CCR8, CCR10, CHRM4, CXCR4, FSHR, GPR103, GPR119, LHCGR, NPSR1b, OXTR, and UTR2.

### Follow-up concentration-response assays for AMB-FUBINACA

Given that initial screening was a single-point assay, we sought to investigate potential hits in detail *via* concentration-response curves. AMB-FUBINACA was selected for further investigation since it had the largest number of strong off-target “hits” at 30 μM (Figure 2). Given the large number of targets, this and subsequent analysis was restricted to targets with a >5-fold difference in S/N and CoV (*n* = 36; mean inhibition 30 μM = 97.00 ± 2.54%). Note also that while GPR119 was inhibited to the greatest extent of any antagonist mode target by several SCRAs, particularly by AMB-FUBINACA, AKB-48, and CUMYL-PINACA (Figure 2), it failed to meet these criteria, potentially related to the relatively high constitutive activity of this receptor (30). Investigated targets spanned 19 families of GPCRs including Class A Orphans, adrenoreceptors, dopamine receptors, chemokine receptors, and urotensin receptors.

**Figure 3:**
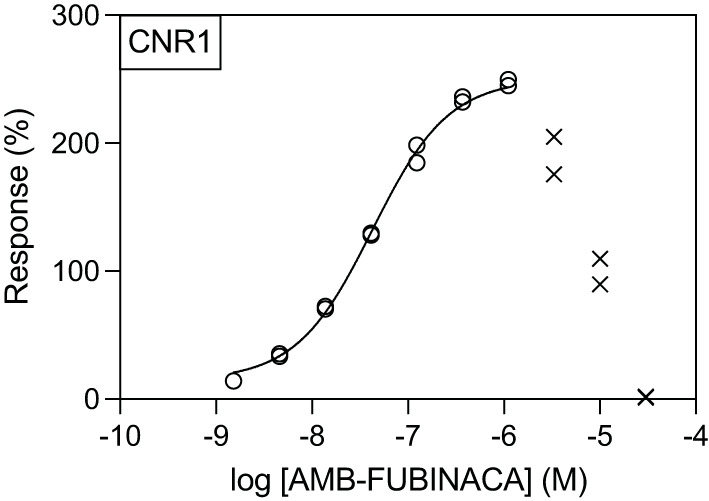
Effect of AMB-FUBINACA in β-arrestin recruitment in CB1-expressing cells. Activity of AMB-FUBINACA is shown with increasing concentration as well as the best fit curve. At concentrations above 1 μM, the response diminished, and this data was excluded from the curve fitting (crosses).

Generation of concentration-response curves against these hits across concentration range of 1.5 nM−30 μM revealed that, unsurprisingly, the greatest potency of AMB-FUBINACA was observed in agonist mode at CB1 (EC_50_ = 42.6 nM; Figure 3). However, the response of CB1-expressing cells to AMB-FUBINACA incrementally decreased above 1 μM, with very little activity remaining at 30 μM.

**Figure 4:**
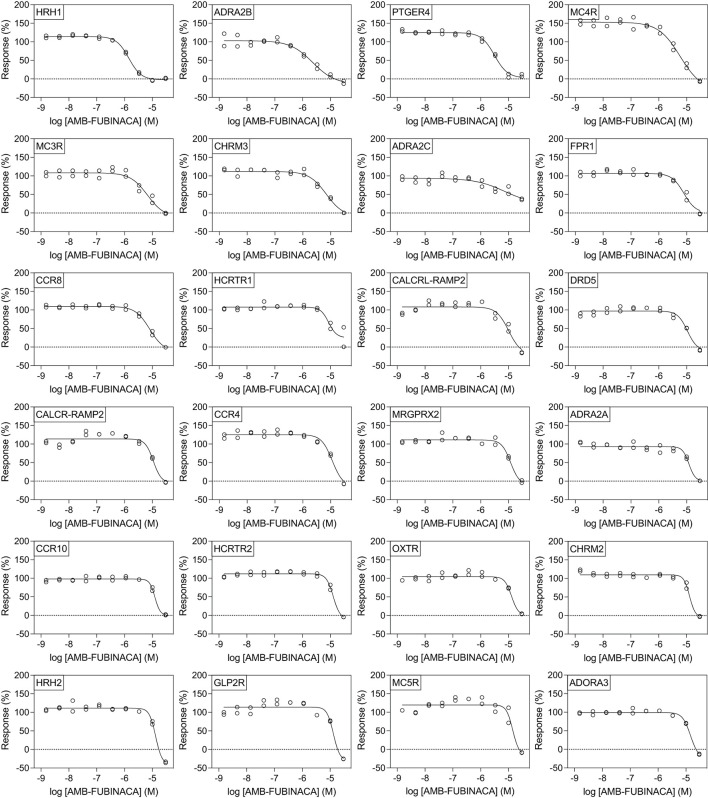
Effect of AMB-FUBINACA in β-arrestin recruitment across 24 GPCRs. Inhibition of AMB-FUBINACA is shown with increasing concentration as well as the best fit curve against EC_80_ of the control ligand at each target (indicated in top left of panel, sorted in order of potency). Control agonists and the respective EC_80_ concentrations used for screening are included as Supplementary Table 2.

**Figure 5:**
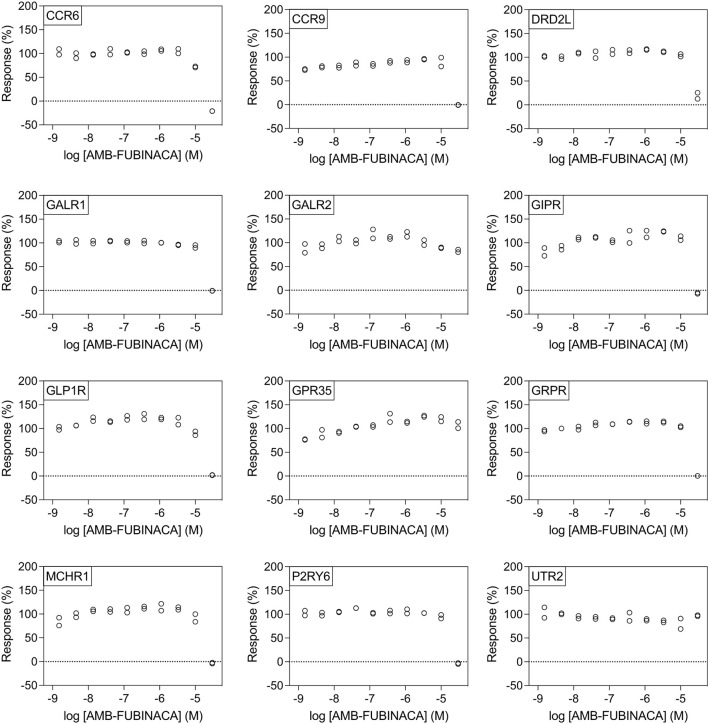
AMB-FUBINACA does not promote potent inhibition of β-arrestin recruitment in 12 GPCRs. IC_50_ of AMB-FUBINACA against each target (listed in the upper left corner of each panel) with EC_80_ agonist could not be determined using concentrations up to 30 μM.

All potencies at other targets were >1 μM (Figure 4), including 12 receptors with where IC_50−_ was >30 μM (Figure 5). The most potent antagonist activity was at HRH1, followed by ADRA2B, PTGER4, MC4R, MC3R, CHRM3, ADRA2C, FPR1, CCR8, and HCRTR1, respectively. Two receptors showed submaximal inhibition, ADRA2C (maximum inhibition = 63.11%) and HCRTR1 (maximum inhibition = 73.00%). Activity was only observed at the highest concentration (30 μM) in several cases (CCR6, CCR9, DRD2L, GALR1, GIPR, MCHR1, and P2RY6), while AMB-FUBINACA did not produce any clear activity at GALR2, GPR35, and UTR2 despite an initial hit in the 30 μM single-point screen.

### AMB-FUBINACA cytotoxicity assay

AMB-FUBINACA cytotoxicity was assayed to probe if the decreasing response of CB1-expressing cells observed at high AMB-FUBINACA concentrations (Figure 3) was the result of cell death.

**Figure 6:**
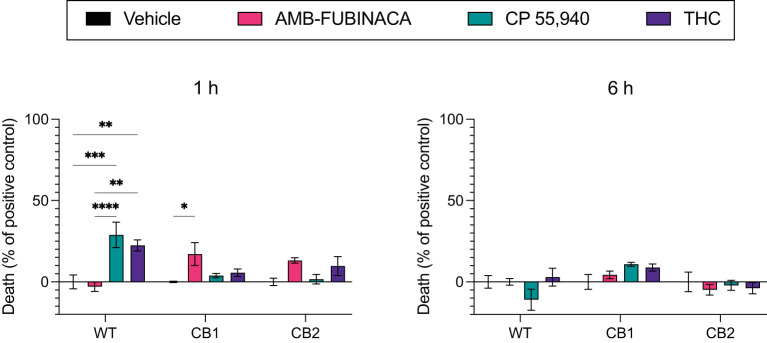
LDH release over time after incubation with cannabinoids. Vehicle, AMB-FUBINACA, CP 55,940, or THC was bath applied to AtT20 cells expressing empty vector (WT), human CB1 (CB1), or human CB2 (CB2). LDH presence in cell media at 1 and 6 h after incubation (as indicated above each graph) was measured, and normalized to total LDH (100%). *n* = 3; two-way ANOVA with Tukey's multiple comparisons test within cell types, at each time point; * = *p* > 0.05, ** = *p* > 0.01; *** = *p* > 0.001; **** = *p* > 0.0001. See Supplementary Table 3 for overall ANOVA results.

Lactate dehydrogenase (LDH) is a cytosolic enzyme and its presence in cellular media is used as a signal of disrupted membrane integrity, and thus often used as a marker of cytotoxicity (31). LDH release was measured after incubation with vehicle, 30 μM AMB-FUBINACA, or two other CB1 receptor agonists, CP 55,940 or THC (30 μM), and compared with total cellular LDH. AMB-FUBINACA produced a small, but significant, increase in the presence of LDH compared with vehicle after a 1 h incubation in CB1 (0.00 ± 0.42 and 17.09 ± 7.03%, respectively; *p* = 0.036; Figure 6), but not WT or CB2 cells (*p* > 0.05). This effect was not observed at 6 h. In contrast, increases in LDH were also measured after incubation with either THC or CP 55,940 in WT cells at 1 h (0.00 ± 4.30, 22.40 ± 3.44, and 28.90 ± 7.81%, in vehicle, THC and CP 55,940, respectively), but no effect was observed at 6 h. This may suggest some off-target activity of CP 55,940 and THC that is not shared with AMB-FUBINACA; however, investigation of this is beyond the scope of the current work.

## Discussion

The off-target activity profiles of seven SCRAs (AMB-FUBINACA, XLR11, PB-22, AKB-48, AB-CHMINICA, CUMYL-PINACA, and 4F-MDMB-BUTINACA) were assessed using the PathHunter β-arrestin assay, with subsequent interrogation of the activity of AMB-FUBINACA against several off-target hits. Overall, there were few hits in agonist mode at targets beyond CB1 and CB2 receptors, but many hits in antagonist mode against a wide range of targets. These hits included the oxytocin receptor and chemokine receptors, among many others, indicating substantial inhibitory promiscuity at 30 μM.

However, literature exploration of these hits revealed few obvious toxigenic targets, with most having either well-characterized and well-tolerated antagonists, or known non-toxic knockout phenotypes (Table 1). In some cases the situation was more complex; for instance, one “hit” was HCRTR1 (orexin OX1 receptor), where some KOs are more susceptible to pharmacologically-induced seizures (32); however, pharmacological inhibition of the receptor does not appear to have the same effect (33). Indeed, the relationship between the OX1 receptor and seizures is multifaceted, such that OX1 antagonists are being investigated for their therapeutic potential in some types of epilepsy (34), thus, it is not known whether or how this susceptibility interacts with CB1-receptor mediated seizures. Moreover, follow-up concentration response experiments using AMB-FUBINACA revealed low potency at this target (IC_50_ 8.59 μM; Figure 4).

**Table 1:** Possible inhibitory off-targets of AMB-FUBINACA.

**Target**	**Common name**	**Family**	**IC_50_ (μM)**	**GTPDB**	**CHEMBL**	**MGI**	**Known antagonists**	**Known KOs**	**Receptor function^#^**
HRH1	H1	Histamine	1.42	262	231	107619	Approved drugs	y	Smooth muscle contraction, neurotransmission
ADRA2B	α2B-adrenoceptor	Adrenoceptors	2.29	26	1942	87935	Approved drugs	y	Adrenergic signaling
PTGER4	EP4	Prostanoid	3.13	343	1836	104311	Tool	y	Prostaglandin signaling
MC4R	MC4	Melanocortin	5.85	285	259	99457	Tool	y	Energy homeostasis and somatic growth
MC3R	MC3	Melanocortin	6.68	284	4644	96929	Tool	y	Energy homeostasis
CHRM3	M3	Acetylcholine	6.96	15	245	88398	Approved drugs	y	Acetylcholine signaling
ADRA2C	α2C-adrenoceptor	Adrenoceptors	7.3	27	1916	87936	Approved drugs	y	Adrenergic signaling
FPR1	FPR1	Formylpeptide	7.91	222	3359	107443	Approved drugs^*^	y	Chemotaxis
CCR8	CCR8	Chemokine	7.99	65	4596	1201402	Tool	y	Chemotaxis
HCRTR1	OX1	Orexin	8.59	321	5113	2385650	Approved drugs	y	Feeding behavior. KOs have increased susceptibility to pharmacologically induced seizures
CALCRL-RAMP2	AM1	Calcitonin	10.1	49	3798	1926944 1859650	Tool (complex)	y	Adrenomedullin signaling. KOs for either Calcrl or Ramp2 are embryonic lethal
DRD5	D5	Dopamine	10.66	218	1850	94927	Approved drugs^*^	y	Dopaminergic signaling
CALCR-RAMP2	AMY2	Calcitonin	10.8	45	2364173	101950 1859650	Tool (CT only)	y	Calcitonin signaling. KOs for either Calcr or Ramp2 are embryonic lethal
CCR4	CCR4	Chemokine	11.48	61	2414	107824	Approved drug	y	Chemotaxis
MRGPRX2	MRGX2	Class A Orphan	11.82	157	5849	3588270 (ortholog)	Tool	y (ortholog)	Mast cell regulation
ADRA2A	α2A-adrenoceptor	Adrenoceptors	12.08	25	1867	87934	Approved drugs	y	Adrenergic signaling
CCR10	CCR10	Chemokine	12.34	67	2321628	1096320	Tool	y	Chemotaxis
HCRTR2	OX2	Orexin	12.74	322	4792	2680765	Approved drugs	y	Feeding behavior, wakefulness
OXTR	OT	Vasopressin and Oxytocin	12.78	369	2049	109147	Approved drugs^*^	y	Neuropeptide signaling
CHRM2	M2	Acetylcholine	12.91	14	211	88397	Approved drugs	y	Acetylcholine signaling
GLP2R	GLP-2	Glucagon	13.09	250	5844	2136733	None	y	Small bowel physiology
HRH2	H2	Histamine	13.09	263	1941	108482	Approved drugs	y	Gastric acid secretion
MC5R	MC5	Melanocortin	14.1	286	4608	99420	Tool	y	Sebum production
ADORA3	A3	Adenosine	14.56	21	256	104847	Approved drugs	y	Adenosine signaling

* Known activity of approved drugs, but not primary mechanism.

# Consult Guide to Pharmacology (GtPdb), Chemogenic European Molecular Biology Laboratory (ChEMBL), and Mouse Genome Informatics (MGI) databases (linked) for detailed descriptions of receptor function.

The follow-up concentration-response experiments demonstrated appreciable inhibitory activity of AMB-FUBINACA at histamine H_1_, and adrenoceptors α2B and α2C, even at lower 1 μM concentrations. Interestingly, H_1_, α2B, and α2C, are all involved in cardiac function, and AMB-FUBINACA has been associated with cardiotoxicity and appears to carry additional risk in people with underlying heart disease (19). However, H_1_, α2B, and α2C antagonists and inverse agonists exist as approved and well-tolerated therapeutic drugs (i.e., common antihistamines, alpha-blockers). Thus, it seems unlikely that these targets are responsible for the cardiotoxicity associated with AMB-FUBINACA. Other targets, such as CCR9, MCHR1, and DRD2L were only inhibited at the highest 30 μM concentration, raising questions concerning the physiological relevance of these ‘hits' for typical SCRA intoxication. Post-mortem tissue analysis in cases where SCRAs have been linked to death has found typical SCRA concentrations <10 ng/mL in blood, occasionally reaching as high as 200 ng/mL (35), corresponding to <25 nM and occasionally up to 500 nM for the SCRAs around 400 g/mol molecular weight, some 2–3 orders of magnitude below our highest 30 μM concentration. A recent study in rats found AMB-FUBINACA concentrations to be ~5-fold higher in brain than plasma, suggesting that brain concentrations may reach as high as 2.7 μM (36).

We selected a high concentration (30 μM) for the initial single-point screen to minimize false-negatives that could occur at lower concentrations (see Figure 4). Nevertheless, it is possible that some false-negatives may have occurred if this high concentration produced cell death or otherwise disrupted cellular signaling. We cannot also rule out the possibility of assay interference due to colloidal aggregation given this concentration (37); however, one would expect to observe pan-inhibition across receptors rather than selected inhibition. This is a fundamental limitation of single-point screens, and it was not feasible to conduct full concentration-response curves for every target with every compound.

Disrupted signaling was evident with medium to high concentrations (>1 μM) of AMB-FUBINACA on CB1-expressing cells (Figure 3). The disrupted signaling could be indicative of CB1-mediated cytotoxicity at high doses; yet the results of our cytotoxicity assay (Figure 6) suggest this is not due to overt cell death. Although LDH release was significantly greater in CB1-expressing cells treated with AMB-FUBINACA, this 18% increase observed only at 1 h does not suggest all the cells are non-viable, and thus does not account for the complete lack of β-arrestin recruitment observed at this concentration. It should be noted, however, that this study was conducted in a different cell type (AtT20 vs. PathHunter CHO cells), and that we only used a single marker of membrane integrity disruption.

Instead, the loss of signal might be explained by a downregulation or degradation of CB1 receptor signaling. Previous studies of investigating β-arrestin recruitment in CB1, including those that have observed recruitment with AMB-FUBINACA, typically have used a much shorter observation window (20 min), with peak β-arrestin-2 recruitment observed with 10 μM AMB-FUBINACA in <10 min (23). Ligand- and concentration-dependent differences in rate of CB1 internalization, particularly at concentrations >10 nM, have also been previously reported (23). Of note, a 15 min application of another potent SCRA, HU210, at 10 nM caused near maximal internalization of CB1, that was not recovered up to 5 h later (38). Encouragingly, we did not observe similar concentration-dependent disruption for any other target selected for follow-up concentration-response experiments with AMB-FUBINACA (Figure 4).

Disrupted signaling of CB1 at high-concentration may be indicative of on-target toxicity for AMB-FUBINACA. CB1 receptor signaling is an integral component of retrograde endocannabinoid inhibition of presynaptic neurons; disabling this retrograde “feedback loop” could produce aberrant neural activity. Seizures have been observed preceding human fatalities involving AMB-FUBINACA (19), and SCRAs can reliably produce convulsant activity in rodent models. Seizures and pro-convulsant neural activity in rodents can be blocked by CB1 receptor antagonist/inverse agonist SR141716 (Rimonabant) or AM-251 (39, 40), which also restores surface CB1 expression following agonist-induced internalization (38). Peripherally, HU-210-induced cardio-depression can be similarly reversed by SR141716 in rats (41), suggesting CB1 receptor involvement, which is potentially of relevance to the bradycardia and hypotension observed during SCRA intoxication in humans (42).

Thus, although we set out to investigate off-target, non-cannabinoid receptor mediated toxicity, the present dataset highlights that high potency and efficacy, to the point of disabling CB1 receptor signaling at high concentrations, may be the predominant GPCR-mediated pathway underpinning the SCRA toxidrome. The underlying cellular mechanism(s), such as receptor internalization, recruitment of β-arrestins, or other signaling modifications, remain to be fully elucidated.

While we investigated the widest range of GPCRs in any SCRA study to date, other receptor classes like ion channels remain to be explored. For instance, several indole- and indazole-carboxamide type SCRAs are appreciably similar in structure to the 5-HT_3_ receptor antagonists alosetron and granisetron. JWH-030, an early-generation partial CB1 and CB2 receptor agonist, inhibits hERG channel current, albeit with relatively low affinity, and may prolong cardiac QT intervals in rats, leading to arrhythmia (43). Moreover, some putative SCRAs related to MEPIRAPIM inhibit voltage-gated T-type calcium (Ca_V_3) channels (44). Other actions such as enzyme induction and inhibition also require further consideration and investigation, particularly in combination with common medications that may be susceptible to adverse pharmacokinetic interactions. Numerous SCRAs of varying subclasses possess inhibitory activity at several cytochrome P450 and glucuronosyltransferase enzymes (45). Finally, other modes of toxicity, such as genotoxicity or toxicity arising from repeated dosing (46–49), and the potential pharmacodynamic and pharmacokinetic contributions of SCRA metabolites or thermal degradants (24, 50), are not encapsulated by the present study.

In sum, broad single-point screening of 7 representative SCRAs against 168 G-protein coupled receptors, with follow-up concentration-response investigations using AMB-FUBINACA, failed to reveal any obvious, standout off-targets that are likely to readily produce toxicity matching the SCRA toxidrome, particularly at concentrations observed in post-mortem tissue. Conversely, a substantial decrease in CB1-mediated signaling, but not overt cytotoxicity, was observed at high concentrations of AMB-FUBINACA, suggesting an on-target mechanism of toxicity whereby cannabinoid receptor signaling is impaired. Nevertheless, other large classes of potential off-targets are yet to be systematically investigated (e.g., ion channels), and toxicity arising from pharmacokinetic interactions or from the route of administration are potential contributing factors that require further investigation.

## Data availability statement

The original contributions presented in the study are included in the article/Supplementary material, further inquiries can be directed to the corresponding author.

## Author contributions

EC, RB, RK, and SB performed and interpreted data analysis. RB carried out the cytotoxicity experiments. RK, EC, and SB drafted the manuscript. All authors contributed to the conception, design of the experimental work, and critically appraised and revised the manuscript.
